# Respiration Can Trigger Cerebrovascular Reactivity: A Novel Method to Quantify Cerebrovascular Resistance Dynamics Using Real‐Time Phase‐Contrast MRI

**DOI:** 10.1002/mrm.70322

**Published:** 2026-02-25

**Authors:** Pan Liu, Qiuting Wen, Kimi Owashi, Jean‐Marc Constans, Cyrille Capel, Olivier Balédent

**Affiliations:** ^1^ Medical Image Processing Department CHU Amiens‐Picardie University Hospital Amiens France; ^2^ CHIMERE (INSERM UA21) Jules Verne University of Picardie Amiens France; ^3^ Department of Radiology and Imaging Sciences Indiana University School of Medicine Indianapolis Indiana USA; ^4^ Radiology Department CHU Amiens‐Picardie University Hospital Amiens France; ^5^ Neurosurgery Department CHU Amiens‐Picardie University Hospital Amiens France

**Keywords:** cerebrovascular reactivity, real‐time phase‐contrast MRI, respiratory effects, respiratory‐band hemodynamics

## Abstract

**Purpose:**

To directly quantify respiration‐driven cerebrovascular resistance dynamics (CRD) using a gas‐free MRI framework based on the internal‐to‐external artery carotid flow ratio (ratio_Q).

**Theory and Methods:**

Two independent datasets (*n* = 10 and *n* = 17) were used to evaluate repeatability and to compare breathing conditions. Real‐time phase‐contrast MRI (RT‐PC) was performed at the C2–C3 level, and a dedicated in‐house software was used to extract the respiratory‐frequency components of internal and external carotid artery flow (Q_ICA and Q_ECA). The flow ratio (ratio_Q = Q_ICA/Q_ECA) was derived to attenuate cardiac‐driven inflow effects. Its mean value (aver_ratio_Q) reflected baseline cerebrovascular resistance, while its pulsatility index (PI_ratio_Q = amplitude/mean) quantified respiration‐driven CRD.

**Results:**

In Dataset 1, ratio_Q showed excellent within‐session repeatability for its mean value (ICC(3,1) = 0.96) and good repeatability for its pulsatility index (ICC(3,1) = 0.79). In Dataset 2, sustained deep breathing increased mean cerebrovascular resistance, evidenced by a decrease in aver_ratio_Q from 2.5 ± 0.9 to 1.7 ± 0.9. Specifically, aver_Q_ICA decreased by 30% (*p* < 0.001), whereas aver_Q_ECA showed no significant change (a 5% increase, *p* = 0.094). Respiration‐driven CRD was enhanced during sustained deep breathing, with PI_ratio_Q increasing by ≈69% compared with free breathing (from 13.6 ± 5.0 to 23.0 ± 7.3, *p* < 0.001).

**Conclusion:**

This gas‐free RT‐PC approach using the ICA‐to‐ECA flow ratio provides a stable and reproducible index of respiration‐driven CRD, enabling isolation of cerebrovascular resistance modulation from cardiac effects and supporting its potential role in the development of faster and more accessible CVR assessment strategies.

## 1 Introduction

Cerebrovascular reactivity (CVR) describes the dynamic adjustment of cerebral vessels to physiological stimuli and helps maintain stable cerebral perfusion [1, 2]. Conventional CVR protocols rely on externally imposed stimuli—hypercapnic CO_2_ inhalation [3], breath‐hold maneuvers [4, 5, 6], or pharmacologic vasodilators [7, 8]—and assess responses using cerebral blood flow‐sensitive readouts (transcranial Doppler [9], arterial spin labeling [10], phase‐contrast MRI [11]) or cerebral blood volume (CBV)‐sensitive readouts (BOLD, VASO) [12, 13]. However, these approaches primarily characterize vascular reactivity to imposed stimuli and do not explicitly address the intrinsic, time‐resolved behavior of cerebrovascular resistance under spontaneous physiological conditions. In particular, whether quiet, spontaneous respiration elicits measurable changes in global cerebrovascular resistance at the respiratory frequency (≈0.15–0.35 Hz) and how this can be quantified without exogenous stimuli remain unclear.

A central methodological challenge lies in disentangling the respective contributions of respiration‐driven cerebrovascular resistance dynamics (CRD) and respiration‐associated modulation of cardiac output mediated by intrathoracic pressure changes, both of which contribute to respiratory‐band fluctuations in cerebral inflow [14, 15, 16]. As a result, flow fluctuations observed at the respiratory frequency cannot be unambiguously attributed to changes in cerebrovascular resistance when cardiac contributions are not controlled or removed. In addition, BOLD‐based measures under respiration can be influenced by susceptibility and motion effects and are further constrained by limited temporal resolution, complicating physiological attribution [1, 5, 17, 18]. Taken together, current CVR methodologies provide limited insight into whether respiration itself induces dynamic changes in global cerebrovascular resistance, a question that is critical for the interpretation and future development of CVR paradigms.

To address this gap, we introduce the concept of CRD, defined here as the temporal fluctuations of global cerebrovascular resistance occurring over physiological timescales. CRD represents a fundamental component of cerebrovascular regulation and is expected to contribute to CVR measured using conventional gas‐challenge paradigms, yet they remain largely unexplored due to limitations in temporal resolution and physiological specificity of existing techniques.

Here, we applied real‐time phase‐contrast MRI (RT‐PC) to quantify respiration‐driven CRD by simultaneously measuring internal and external carotid artery (ICA and ECA) flows. By deriving an ICA‐to‐ECA flow ratio (ratio_Q), we suppress global variations in common carotid inflow associated with cardiac output and isolate respiration‐related redistribution of flow that reflects dynamic modulation of cerebrovascular resistance.

This study aims to (i) establish the feasibility and test–retest stability of RT‐PC MRI for quantifying respiration‐driven CRD using ratio_Q, and (ii) examine free versus sustained deep‐breathing conditions to probe the dynamic range of the response and evaluate its physiological specificity at the respiratory frequency. We hypothesize that ratio_Q provides a robust, noninvasive, gas‐free index of respiration‐driven CRD, thereby laying a methodological foundation for future, more time‐efficient and lower‐cost CVR assessment strategies.

## 2 Methods

Preliminary results were presented at the 2025 ISMRM Annual Meeting, Honolulu, USA (Abstract No. 0278) [19]. This manuscript (i) adds an independent cohort for repeatability analysis (Dataset 1, *n* = 10) and (ii) expands the core analysis cohort (Dataset 2) by five participants.

### 2.1 Theory

**FIGURE 1:**
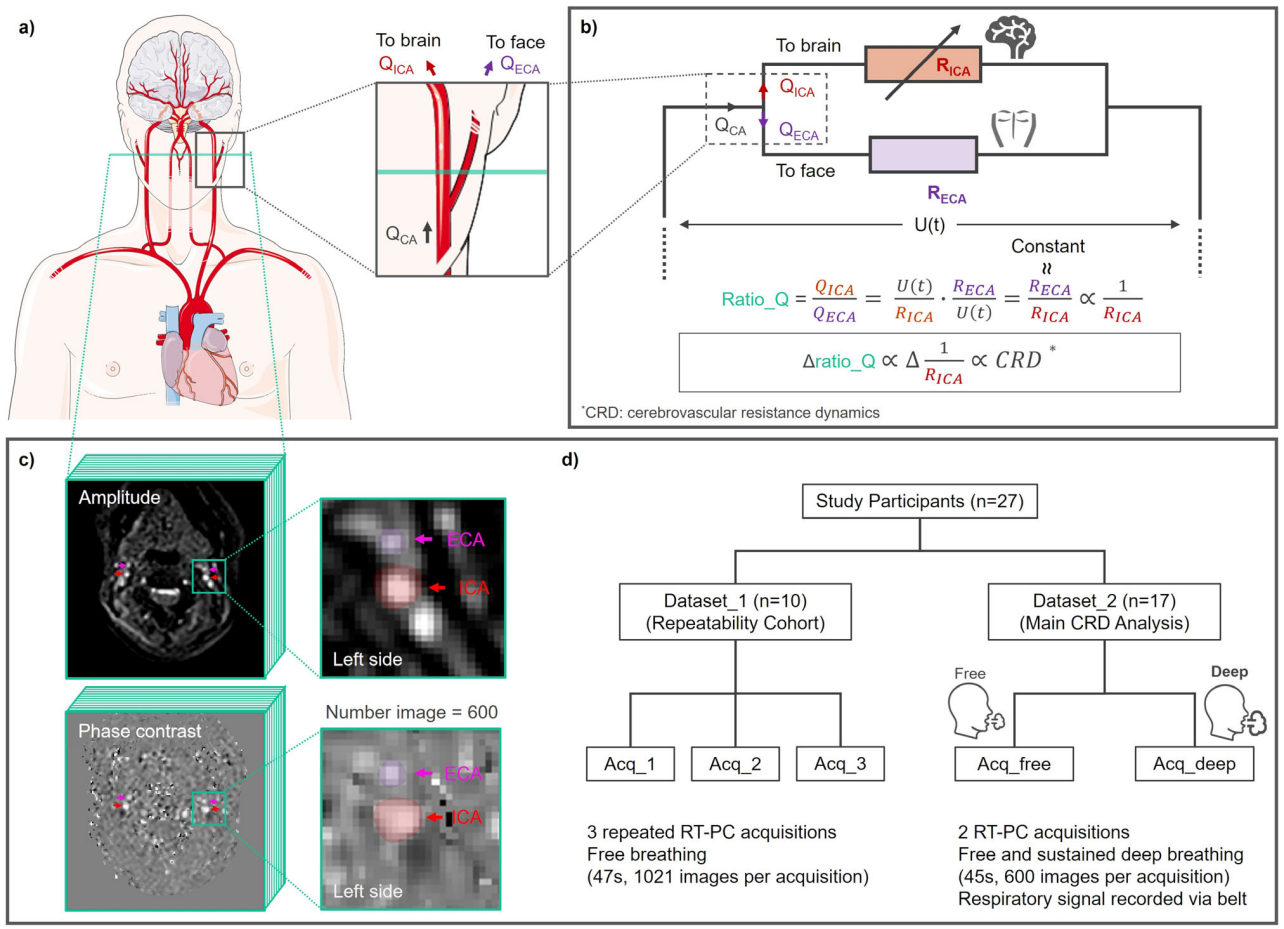
Concept and experimental design for quantifying respiration‐driven cerebrovascular resistance dynamics (CRD) with real‐time phase‐contrast MRI. (a) Measurement schematic at the cervical carotid level: total common carotid inflow (Q_CA) divides into the internal carotid (Q_ICA, red) and external carotid (Q_ECA, purple) branches. (b) Parallel‐circuit model. Respiration‐driven CRD is represented as a dynamic change in internal carotid resistance (R_ICA), while external carotid resistance (R_ECA) is assumed constant. Given a common inflow, the flow ratio (ratio_Q = Q_ICA/Q_ECA) is inversely proportional to R_ICA and therefore reflects CRD. (c) Representative RT‐PC frames showing left‐sided ICA and ECA segmentations on magnitude and phase images. (d) Study design. Dataset 1 (*n* = 10) evaluated test–retest repeatability using three RT‐PC runs during free breathing. Dataset 2 (*n* = 17) assessed respiration‐driven CRD during free breathing and sustained deep breathing. Respiration was recorded synchronously with a belt sensor.

Respiration induces intrathoracic pressure swings that secondarily modulate cardiac output and propagate through the arterial tree. In the extracranial cross‐section, the internal (ICA) and external (ECA) carotid arteries behave as parallel branches subject to a common proximal pressure input (Figure 1a). We model respiration‐driven CRD as a respiration‐modulated change in ICA resistance (R_ICA) while ECA resistance (R_ECA) is assumed relatively stable under controlled temperature and resting conditions [20, 21]. Under a common inflow, the flow ratio (ratio_Q = flow ratio of ICA and ECA) varies inversely with R_ICA and emphasizes respiration‐related redistribution of flow between ICA and ECA, thereby attenuating confounds from global cardiac output fluctuations (Figure 1b). Concretely, when R_ICA increases (vasoconstriction), Q_ICA decreases relative to Q_ECA and ratio_Q falls; when R_ICA decreases (vasodilation), Q_ICA increases relative to Q_ECA and ratio_Q rises. On this basis, ratio_Q is used as the primary index of respiration‐driven CRD.

### 2.2 Participants

Two cohorts of healthy volunteers were included: Dataset_1 (*n* = 10; mean age, 50 ± 11 years; range, 36–72 years; 6 males) and Dataset_2 (*n* = 17; mean age, 29.6 ± 9.5 years; range, 19–54 years; 11 males). Exclusion criteria were MRI contraindications, history of cerebrovascular or respiratory disease, or abnormal screening MRI. All participants provided written informed consent.

The study protocols were approved by the Institutional Review Board of Indiana University (Dataset 1) and the Comité de Protection des Personnes Nord Ouest II, Amiens University Hospital (Dataset 2). All procedures complied with the Declaration of Helsinki.

### 2.3 MRI Acquisition

Imaging was performed with participants in the supine position on 3 T scanners equipped with 32‐channel head coils. Dataset 1 and Dataset 2 were acquired independently at two institutions using different 3 T MRI systems (Dataset 1: Siemens Prisma, maximum gradient = 80 mT/m; slew rate = 200 mT/m/ms; Dataset 2: Philips Achieva, maximum gradient = 80 mT/m; slew rate = 120 mT/m/ms). Each site optimized acquisition parameters according to scanner‐specific gradient performance by first ensuring sufficient temporal resolution (< 100 ms) to accurately capture cardiac‐frequency flow amplitudes, which is particularly critical for highly pulsatile arterial flow [14]. Within this constraint, spatial resolution was further maximized to reduce partial volume effects (Figure 1c) [22, 23]. For Dataset_2, respiration signal was recorded synchronously with a belt sensor (Figure 1d). The two datasets served distinct purposes and were analyzed separately, with no cross‐use or pooling.

Both cohorts used a 2D EPI‐based real‐time phase‐contrast sequence with a Cartesian k‐space trajectory; positive velocity was defined cranially (caudal‐to‐cranial).

Dataset_1 (used for repeatability; free breathing): shared velocity‐encoding (+M1/−M1); FOV 240 × 240 mm^2^; voxel 1.1 × 1.1 mm^2^; slice 10 mm; VENC 70 cm/s; temporal resolution 47 ms; 1024 frames over 47 s; flip angle 10°.

Dataset_2 (used for CRD analysis; free breathing and sustained deep breathing): two consecutive acquisitions—one during free breathing and one during sustained deep breathing performed at a self‐paced rhythm. Conventional velocity‐encoding (flow‐encoded/flow‐compensated); FOV 140 × 140 mm^2^; voxel 2 × 2 mm^2^; slice 4 mm; VENC 60 cm/s; temporal resolution 75 ms; 600 frames over 45 s; flip angle 10°. The accuracy of flow quantification at these spatial/temporal resolutions has been validated previously [14].

### 2.4 Image and Signal Post‐Processing and Parameter Extraction

Image and signal post‐processing were performed using *Flow 2.0*, a noncommercial software developed in IDL (Interactive Data Language) [24].

**FIGURE 2:**
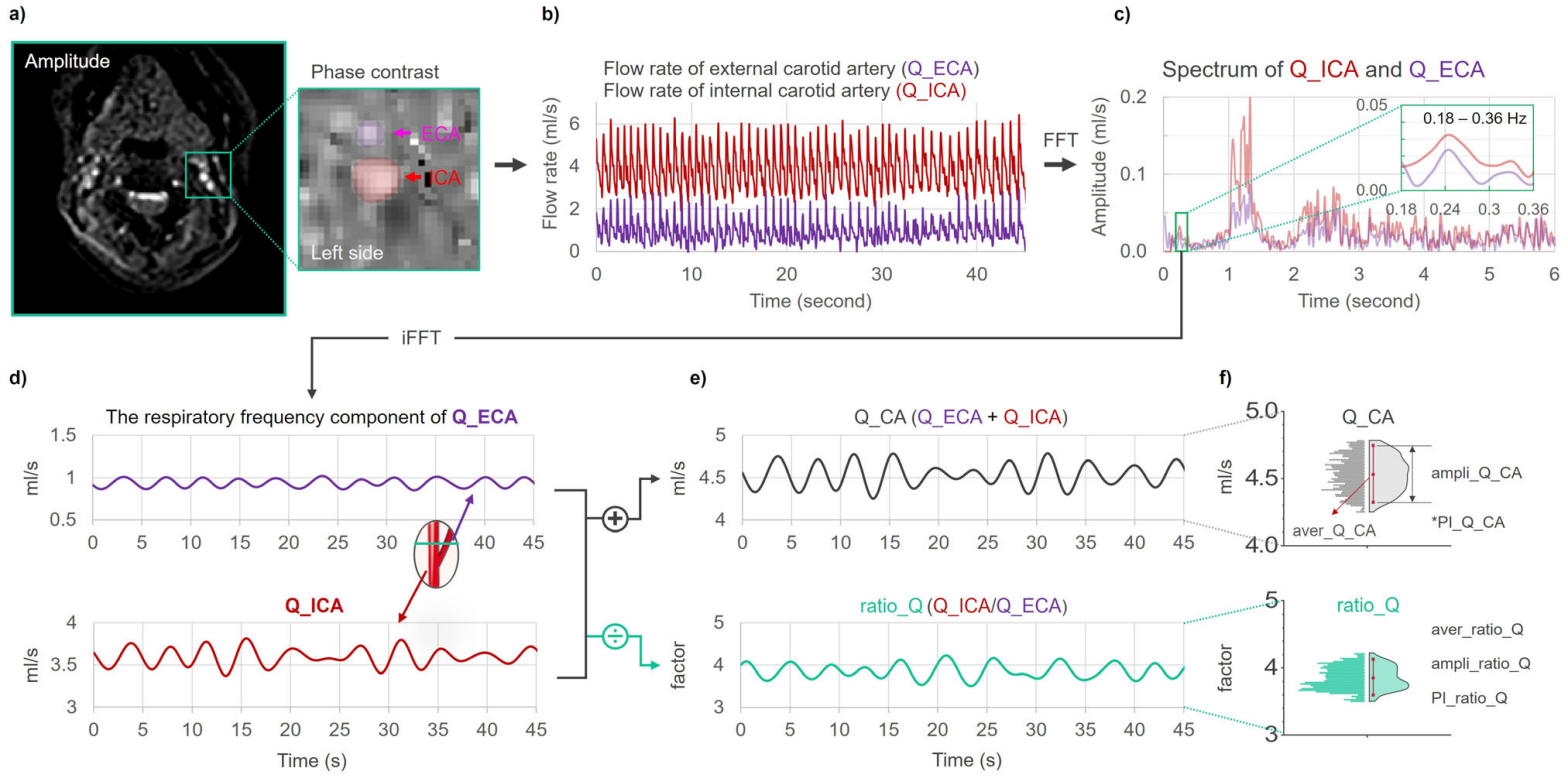
Processing workflow to derive respiration‐band flow dynamics and CRD‐related metrics from RT‐PC. (a) Vessel segmentation and background‐phase correction using dedicated RT‐PC post‐processing. (b) Extraction of carotid flow‐rate time series for the internal (Q_ICA) and external (Q_ECA) carotid arteries (units: mL/s). (c) Fast Fourier transform (FFT) to obtain spectra and identify the respiratory peak within 0.18–0.36 Hz in this example. (d) Band‐limited signals at the respiratory frequency reconstructed by applying a narrow bandpass in the Fourier domain followed by inverse FFT. (e) Computation of total common carotid flow (Q_CA = Q_ICA + Q_ECA) and the flow ratio ratio_Q = Q_ICA/Q_ECA (dimensionless). (f) Quantitative metrics: mean (aver_Q_CA, aver_ratio_Q); amplitude (ampli_Q_CA, ampli_ratio_Q; defined as the 5th–95th percentile range); and pulsatility index (PI_Q_CA, PI_ratio_Q), where PI = amplitude/mean.

Image post‐processing consisted of the following four steps:

1. Dynamic ROI segmentation. Arterial ROIs (ICA, ECA) were automatically identified and tracked frame‐by‐frame with constant area and centroid tracking to follow vessel motion (Figure 2a).
2. Background phase correction. Stationary tissue around each vessel was automatically detected and its mean velocity subtracted from each frame to remove background phase offsets
3. Velocity unwrapping. Phase aliasing due to velocities exceeding VENC was corrected with a temporal unwrapping algorithm.
4. Flow‐rate calculation. Q_ICA and Q_ECA (mL/s) were computed as ROI area × mean velocity within the ROI (Figure 2b).

Signal post‐processing included three steps:

1. Respiratory‐band definition. The subject‐specific respiratory peak was identified from the internal jugular vein (IJV) flow‐rate trace acquired in the same slice for Dataset_1, this is because venous flow exhibits larger‐amplitude respiratory oscillations and a stronger respiratory‐band component than arterial flow [14, 25], making it a more reliable reference for respiratory‐band identification. For Dataset 2, the respiratory band was defined from the belt‐recorded respiratory signal.
  Specifically, the IJV flow signal or respiratory belt signal was transformed into the frequency domain using fast Fourier transform (FFT). The respiratory band was then defined as an approximately 0.16‐Hz‐wide frequency range centered on the dominant respiratory component, with minor adjustments to account for inter‐individual variability in respiration period (Figure 2c).
2. Internal and external carotid artery flow signals (Q_ICA and Q_ECA) were transformed into the frequency domain using FFT. Only the complex spectral components within the predefined respiratory band were retained, and respiration‐band–limited time‐domain signals were reconstructed using inverse FFT. This procedure is equivalent to a frequency‐domain hard‐window band‐pass filter, which preserves spectral conjugate symmetry and avoids phase distortion. Given the narrow bandwidth of the respiratory frequency range, this approach minimizes contamination from adjacent frequency components (Figure 2d).
3. Composite and ratio. Total common carotid flow Q_CA = Q_ICA + Q_ECA and the flow ratio ratio_Q = Q_ICA/Q_ECA were derived (Figure 2e).

For Q_CA and ratio_Q, to minimize edge effects introduced by frequency‐domain hard‐window filtering, the first and last 3 s of the reconstructed respiration‐band time series were excluded from subsequent analysis. We then computed: aver_Q_CA and aver_ratio_Q (means); ampli_Q_CA and ampli_ratio_Q (robust amplitudes, defined as the 5th–95th percentile range); and PI_Q_CA and PI_ratio_Q (pulsatility index), where PI = ampli_/aver_ (Figure 2f).

Because inter‐individual anatomical differences introduce variability in the baseline level of ratio_Q, and breathing patterns can substantially modulate the amplitude of respiratory‐frequency oscillations in cerebral arterial flow [26], direct comparisons based solely on absolute respiratory‐band amplitudes (ampli_ratio_Q) may be unreliable. To mitigate these effects and enable robust comparisons across subjects and breathing conditions, we therefore relied primarily on the normalized pulsatility index (PI = ampli_/aver_). PI_Q_CA indexes respiration‐driven fluctuations in total carotid inflow, whereas PI_ratio_Q quantifies the normalized magnitude of respiration‐driven CRD.

### 2.5 Intra‐Session Repeatability Assessment

Participants in Dataset_1 underwent three consecutive RT‐PC runs under identical conditions within a single session to evaluate within‐session repeatability and potential run‐to‐run drift. Agreement for all quantitative parameters (aver_, ampli_, PI) was assessed using the intraclass correlation coefficient, two‐way mixed‐effects model, absolute agreement, single‐measurement—ICC(3,1). Interpretation followed conventional thresholds: poor (< 0.50), moderate (0.50–0.75), good (0.75–0.90), and excellent (> 0.90). Statistical analyses were performed in SPSS v26 (IBM).

### 2.6 Respiratory‐Cycle‐Based Signal Reconstruction

Respiratory‐cycle analysis was performed on Dataset_2 to examine Q_CA and ratio_Q dynamics under free breathing and sustained deep breathing.

1. Respiratory cycle detection. The software automatically identified extrema of the respiratory signal as segmentation points, with each cycle corresponding to the period from expiration to inspiration.
2. Segmentation and reconstruction. Using these segmentation points, Q_CA and ratio_Q were divided into individual respiratory cycles, each interpolated to 32 sample points and averaged to reconstruct a mean respiratory‐cycle waveform.

**FIGURE 3:**
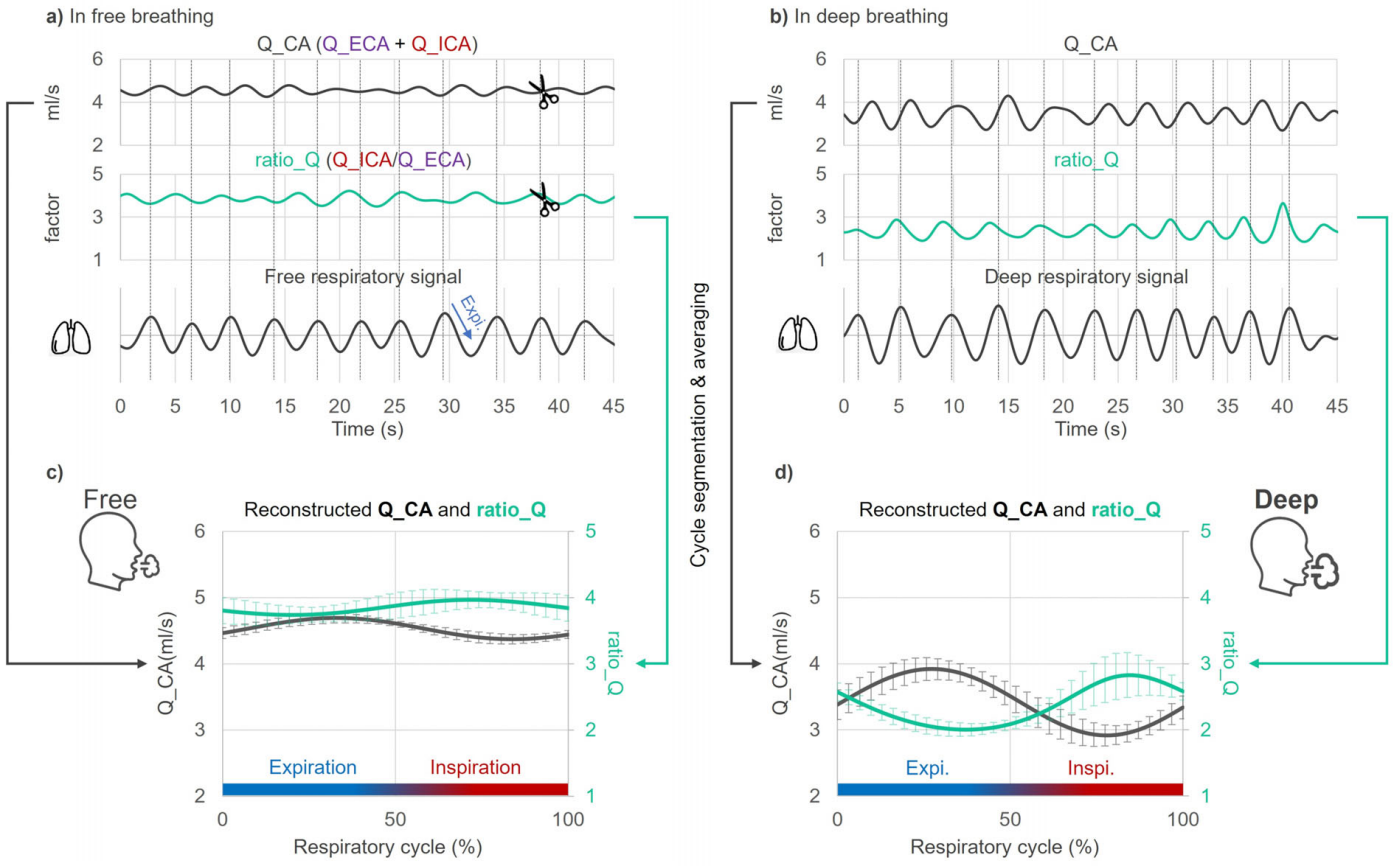
Reconstruction of respiratory‐cycle waveforms of Q_CA and ratio_Q. (a, b) Raw time series of total common carotid flow (Q_CA, black; left *y*‐axis, mL/s) and flow ratio ratio_Q (Q_ICA/Q_ECA, cyan; right *y*‐axis, dimensionless). Respiratory cycles were segmented using peak detection of the belt signal under (a) free breathing and (b) sustained deep breathing. (c, d) Cycle‐averaged waveforms (0%–100% of the cycle) for Q_CA and ratio_Q for (c) free breathing and (d) sustained deep breathing. In both conditions, Q_CA shows phase‐locked oscillations that peak during expiration. The falling edge of ratio_Q occurs near the Q_CA peak and corresponds to an increase in cerebrovascular resistance (higher R_ICA). Error bars denote within‐subject standard deviation across cycles. Expiration and inspiration are indicated in blue and red, respectively.

This method was applied solely to compare respiration‐locked variations in Q_CA and ratio_Q between breathing conditions and to assess their relative phase (Figure 3).

### 2.7 Statistical Analysis

Nonparametric tests were used a priori. Paired comparisons employed the Wilcoxon signed‐rank test (two‐tailed), and unpaired comparisons the Mann–Whitney *U* test (Wilcoxon rank‐sum; two‐tailed). Associations between variables were assessed with Spearman's rank correlation (ρ). Repeatability was quantified with ICC(3,1) as detailed in Intra‐Session Repeatability Assessment. Statistical significance was set at *p* < 0.05. Analyses were performed in SPSS v26 (IBM), and figures in Origin 2022 (OriginLab). Data are reported as mean ± SD and interquartile range [Q1–Q3] (25th–75th percentile).

## 3 Results

All scans were analyzable (Dataset_1: *n* = 10; Dataset_2: *n* = 17).

### 3.1 Intra‐Session Repeatability (Dataset_1)

Mean flow rates showed the highest within‐session repeatability, with all ICC(3,1) values classified as Excellent (≥ 0.90). In contrast, PI_Q_ICA, PI_Q_ECA, and PI_Q_CA yielded Moderate repeatability (ICC 0.64–0.72).

Among CRD‐related metrics, aver_ratio_Q was Excellent (ICC = 0.96), whereas PI_ratio_Q reached Good repeatability (ICC = 0.79; 95% CI 0.62–0.90). Averaging three runs further improved agreement—ICC(3,k) reached Good–Excellent for all parameters (Table 1).

**TABLE 1:** Intra‐session repeatability of hemodynamic and CRD‐related parameters derived from RT‐PC MRI.

Parameter	Mean ± SD	ICC(3,1)	95% CI	Interpretation*	ICC(3,k)	95% CI	Interpretation*
aver_Q_ICA (mL/s)	4.2 ± 0.9	0.99	0.98–0.99	Excellent	0.99	0.99–0.99	Excellent
aver_Q_ECA (mL/s)	1.9 ± 0.6	0.97	0.94–0.99	Excellent	0.99	0.98–0.99	Excellent
aver_Q_CA (mL/s)	6.2 ± 1.2	0.99	0.97–0.99	Excellent	0.99	0.99–0.99	Excellent
ampli_Q_CA (mL/s)	0.5 ± 0.1	0.72	0.52–0.87	Moderate	0.89	0.76–0.95	Good
PI_Q_ICA (%)	7.4 ± 2.3	0.68	0.46–0.85	Moderate	0.87	0.72–0.94	Good
PI_Q_ECA (%)	14.8 ± 5.0	0.72	0.52–0.87	Moderate	0.89	0.76–0.95	Good
PI_Q_CA (%)	7.8 ± 2.2	0.64	0.4–0.82	Moderate	0.84	0.67–0.93	Good
aver_ratio_Q	2.4 ± 0.9	0.96	0.92–0.98	Excellent	0.99	0.97–0.99	Excellent
ampli_ratio_Q	0.34 ± 0.20	0.74	0.54–0.88	Moderate	0.89	0.78–0.96	Good
PI_ratio_Q (%)	14.4 ± 5.5	0.79	0.62–0.90	Good	0.92	0.83–0.97	Good

*Note*: Values are reported as mean ± SD across 60 measurements (10 participants × 2 sides × 3 runs). Intraclass correlation coefficients (ICC) were estimated with a two‐way mixed‐effects model, absolute agreement. ICC(3,1) reflects reliability of a single run; ICC(3,k) reflects reliability of the average of three runs. * Interpretation thresholds: Excellent (≥ 0.90), Good (0.75–0.89), Moderate (0.50–0.74), Poor (< 0.50). Units: mL/s for flow‐rate averages (aver_Q) and ampli_Q of Q_CA; % for PI; dimensionless for aver_ratio_Q and ampli_ratio_Q. ratio_Q, ICA‐to‐ECA flow ratio (Amplitude defined as the 5th–95th percentile range; PI = ampli_/aver_).

Abbreviations: CA, common carotid artery; ECA, external carotid artery; ICA, internal carotid artery.

**FIGURE 4:**
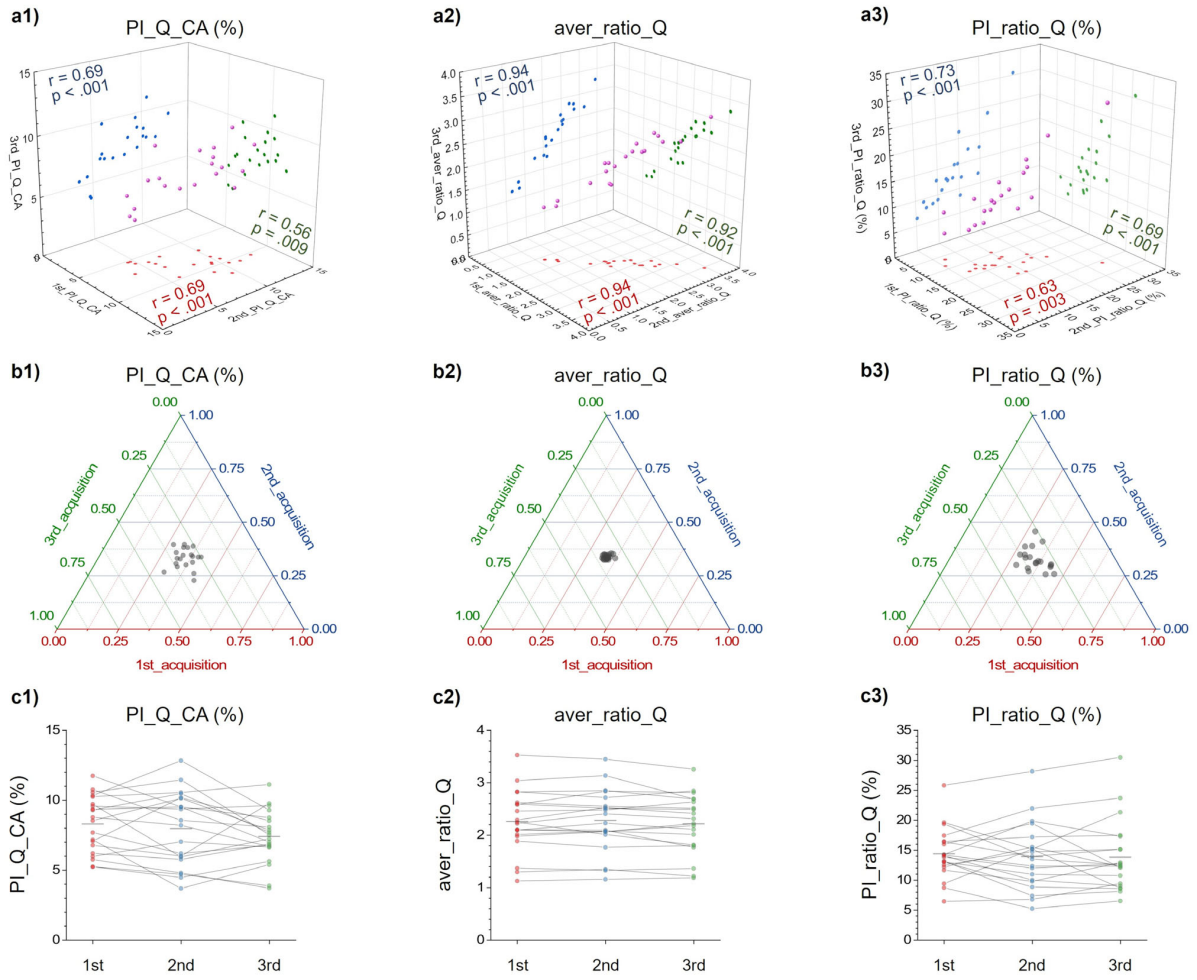
Repeatability of key parameters across three within‐session RT‐PC runs (Dataset_1). Columns show PI_Q_CA (pulsatility index of total carotid flow), aver_ratio_Q (mean ICA/ECA flow ratio), and PI_ratio_Q (pulsatility index of the flow ratio). (a1–a3) 3D scatter plots across the 1st, 2nd, and 3rd runs with corresponding Spearman correlations (reported as *r*) and *p*‐values. (b1–b3) Ternary plots showing the proportional contribution of each run per subject; clustering near the center indicates a consistent distribution among runs. (c1–c3) Subject‐level trajectories across runs. No significant differences were detected among runs by the Friedman test (*p* > 0.05). CA, common carotid artery; ECA, external carotid artery; ICA, internal carotid artery; ratio_Q, ICA‐to‐ECA flow ratio. (PI = ampli_/aver_).

Figure 4 summarizes three representative metrics (PI_Q_CA, aver_ratio_Q, PI_ratio_Q): (a) strong pairwise Spearman correlations across the three runs; (b) ternary plots clustering near center (≈33/33/33), consistent with the ICCs; and (c) subject‐level traces without systematic run‐to‐run drift (Friedman test, all *p* > 0.05).

### 3.2 Condition Effects: Free Versus Sustained Deep Breathing (Dataset_2)

Across conditions, most parameters differed significantly (Table 2). Mean ICA and total inflow (aver_Q_ICA, aver_Q_CA) were lower during sustained deep breathing than free breathing (both *p* < 0.001), whereas mean ECA inflow (aver_Q_ECA) did not change significantly (*p* = 0.094).

**TABLE 2:** Comparison of hemodynamic and CRD‐related parameters between free and sustained deep breathing.

Parameter (*n* = 17 × 2)	Free breathing	Deep breathing	Deep/Free	*Z* (Wilcoxon)	*p*
Respiratory cycle (s) (Q1–Q3)	3.8 ± 0.7	6.2 ± 2.1	163%	3.41	< 0.001
	(3.5–4.2)	(4.8–6.8)			
aver_Q_ICA (mL/s)	4.3 ± 0.9	3.0 ± 0.7	70%	−5.06	< 0.001
	(3.7–4.6)	(2.5–3.3)			
aver_Q_ECA (mL/s)	2.0 ± 0.9	2.1 ± 0.9	105%	1.68	**0.094**
	(1.4–2.2)	(1.5–2.5)			
aver_Q_CA (mL/s)	6.3 ± 1.4	5.1 ± 1.3	81%	−5.08	< 0.001
	(5.3–6.8)	(4.3–5.6)			
ampli_Q_ICA (mL/s)	0.36 ± 0.14	0.71 ± 0.23	197%	5.04	< 0.001
	(0.26–0.42)	(0.51–0.87)			
ampli_Q_ECA (mL/s)	0.31 ± 0.17	0.59 ± 0.24	190%	4.60	< 0.001
	(0.19–0.36)	(0.43–0.76)			
ampli_Q_CA (mL/s)	0.59 ± 0.22	1.19 ± 0.37	202%	4.94	< 0.001
	(0.42–0.74)	(0.87–1.44)			
PI_Q_ICA (%)	8.5 ± 2.6	25.2 ± 10.5	296%	5.06	< 0.001
	(6.9–10.0)	(16.2–30.7)			
PI_Q_ECA (%)	15.8 ± 4.7	29.4 ± 8.7	186%	4.96	< 0.001
	(11.7–19.1)	(22.4–31.1)			
PI_Q_CA (%)	9.4 ± 2.7	24.1 ± 8.6	256%	5.08	< 0.001
	(7.6–11.2)	(17.1–29.1)			
aver_ratio_Q	2.5 ± 0.9	1.7 ± 0.9	68%	−5.01	< 0.001
	(1.8–3.1)	(1.1–1.9)			
ampli_ratio_Q	0.32 ± 0.14	0.39 ± 0.24	122%	2.02	0.043
	(0.21–0.39)	(0.23–0.50)			
PI_ratio_Q (%)	13.6 ± 5.0	23.0 ± 7.3	169%	4.48	< 0.001
	(10.5–16.3)	(15.8–30.3)			

*Note*: Values are presented as mean ± SD with [Q1–Q3] in parentheses, based on 34 measurements from 17 participants (left and right sides). Condition differences were tested with the Wilcoxon signed‐rank test; Z statistics are reported and *p* < 0.05 was considered significant, bold values indicate non‐significant differences (*p* ≥ 0.05). “Deep/Free (%)” denotes the ratio of the deep‐breathing value to the free‐breathing value ×100. Units: mL/s for flow‐rate averages (aver_Q) and amplitudes (ampli_Q); % for pulsatility indices (PI); dimensionless for aver_ratio_Q and ampli_ratio_Q.

Abbreviations: CA, common carotid artery; ECA, external carotid artery; ICA, internal carotid artery; PI, pulsatility index; PI_ratio_Q, pulsatility index of ratio_Q; Q, flow rate; ratio_Q, ICA‐to‐ECA flow ratio.

**FIGURE 5:**
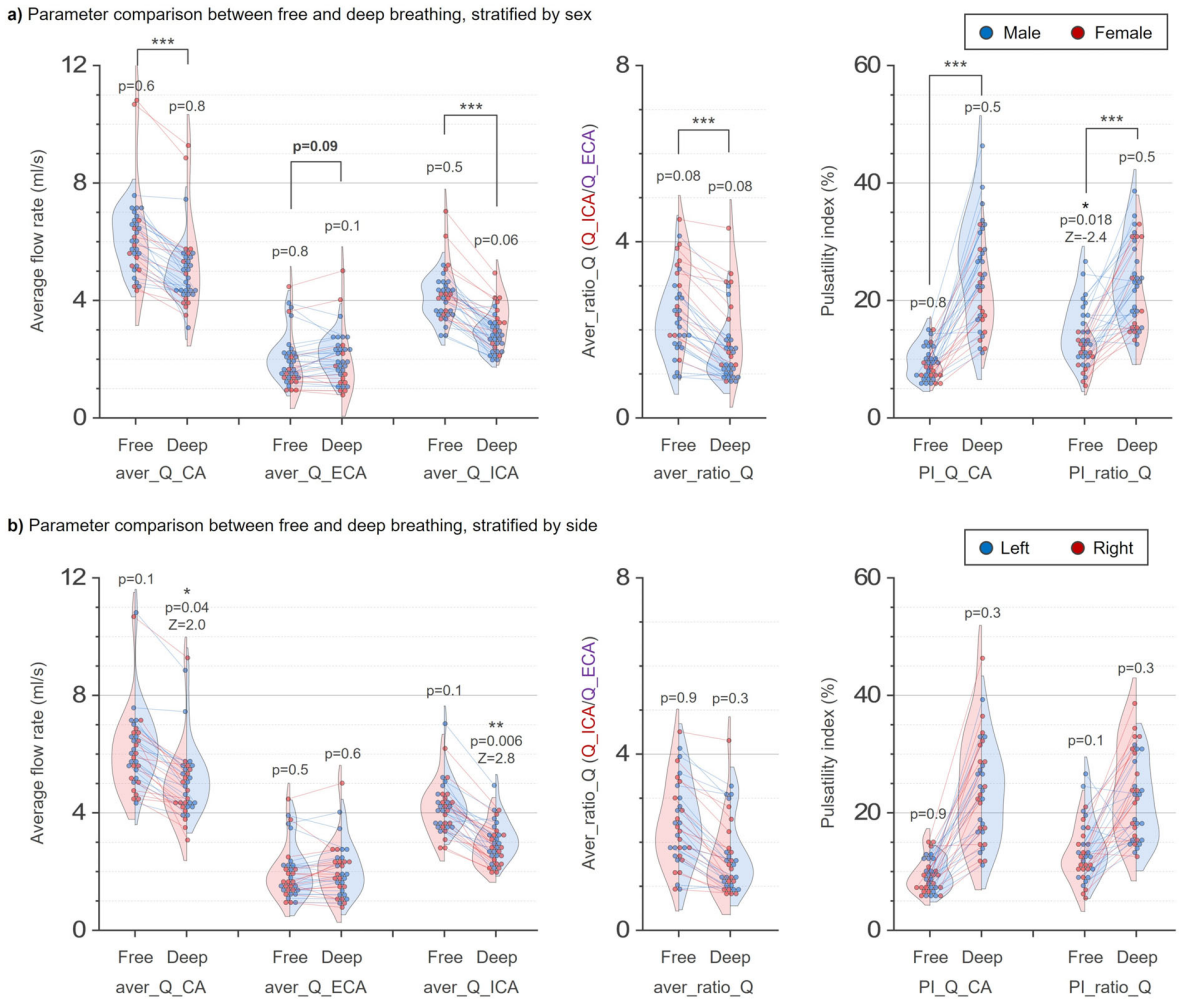
Comparison of key flow parameters between free and deep breathing conditions. (a) Distributions grouped by sex (males: *n* = 11, blue; females: *n* = 6, red) for aver_Q_CA, aver_Q_ECA, aver_Q_ICA, aver_ratio_Q, PI_Q_CA, and PI_ratio_Q. Within‐subject differences between breathing states (*n* = 34 measurements: 17 participants × 2 sides) were tested with the Wilcoxon signed‐rank test; between‐sex differences with the Mann–Whitney *U* test. (b) Corresponding distributions grouped by carotid side (left: blue; right: red). Side‐related differences were evaluated with the Wilcoxon signed‐rank test (paired). Violin plots show the empirical distributions. Panel‐wise *p*‐values are annotated above brackets. CA, common carotid artery; ECA, external carotid artery; ICA, internal carotid artery; PI, pulsatility index; ratio_Q, ICA‐to‐ECA flow ratio.

Respiration‐band waveform metrics increased under sustained deep breathing: amplitudes (ampli_Q_ICA, ampli_Q_ECA, ampli_Q_CA) and pulsatility indices (PI_Q_ICA, PI_Q_ECA, PI_Q_CA) were all elevated (*p* < 0.001 for all). For the carotid flow ratio, aver_ratio_Q decreased (*p* < 0.001), while ampli_ratio_Q and PI_ratio_Q increased (*p* = 0.043 and *p* < 0.001, respectively). Figure 5 visualizes these distributions with paired within‐subject comparisons.

### 3.3 Sex and Side Stratifications (Dataset_2)

In sex‐stratified analyses (Figure 5a), PI_ratio_Q during free breathing was higher in males than females (*p* = 0.018); this difference was not present during sustained deep breathing. No other sex effects were significant.

In side‐stratified analyses (Figure 5b), most parameters were comparable between left and right carotids; the only exception was aver_Q_ICA during sustained deep breathing, which was higher on the left (*p* = 0.006).

### 3.4 Respiratory‐Cycle Waveforms of Q_CA and ratio_Q (Dataset_2)

**FIGURE 6:**
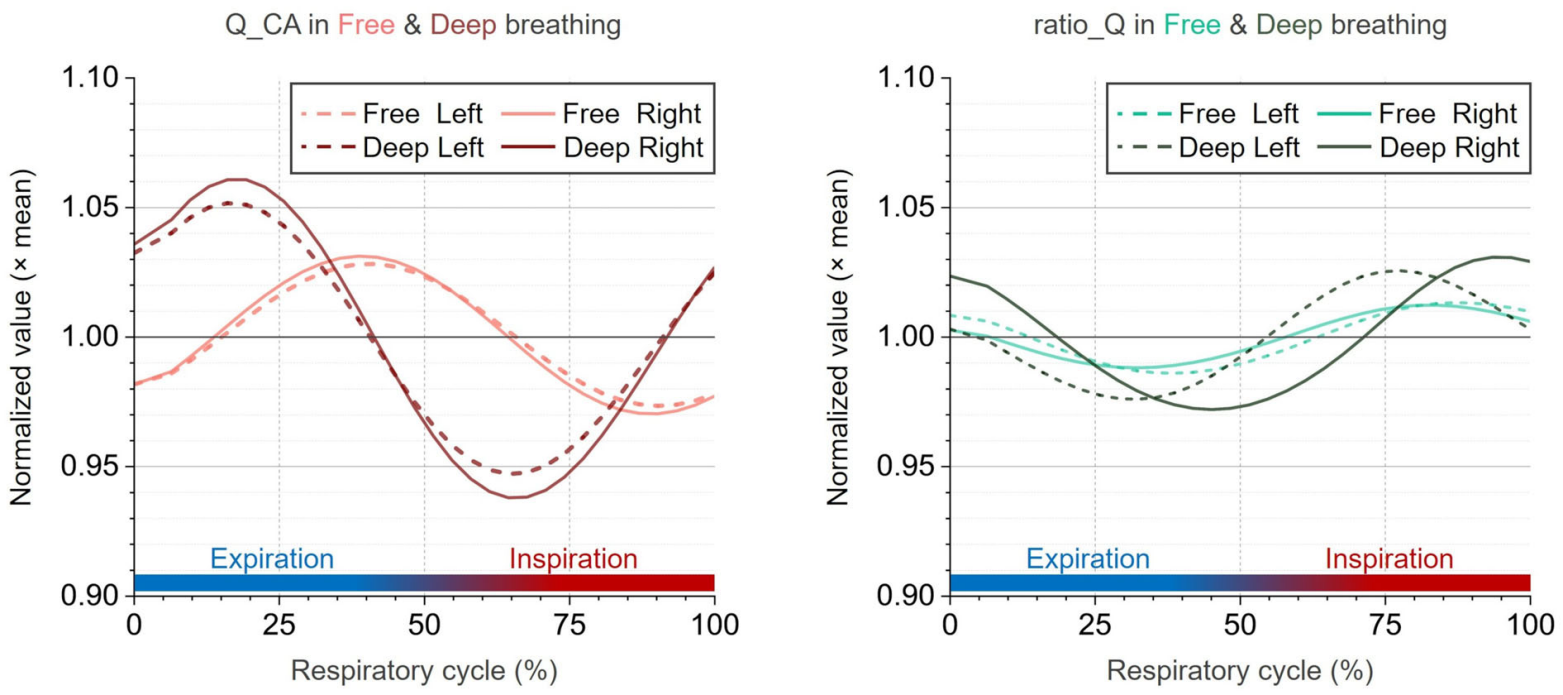
Reconstructed respiratory‐cycle waveforms of Q_CA and ratio_Q during free and deep breathing. Normalized respiratory‐cycle waveforms are shown separately for the left and right sides, with free breathing indicated by dashed lines and deep breathing by solid lines. Left: group‐averaged Q_CA waveforms. Right: group‐averaged ratio_Q waveforms. Light colors denote free breathing, and darker colors denote deep breathing. All signals are normalized to each subject's mean value (dimensionless). The *x*‐axis represents the respiratory cycle (0%–100%), with expiration and inspiration phases indicated.

Reconstructed respiratory‐cycle waveforms (Figure 6) were similar between left and right sides for both breathing modes. Sustained deep breathing produced larger respiration‐locked fluctuations in Q_CA and ratio_Q (consistent with Table 2).

Phase behavior differed by breathing mode. During free breathing, increases in Q_CA coincided with the falling edge of ratio_Q, indicating a transient increase in cerebrovascular resistance (higher R_ICA). During sustained deep breathing, the Q_CA descent shifted earlier (from early expiration into early inspiration), whereas ratio_Q maintained a consistent pattern, increasing during inspiration in both conditions (Figure 6).

Individual reconstructed respiratory‐cycle waveforms and side‐specific variability are shown in Figure S1.

## 4 Discussion

This study introduces a real‐time phase‐contrast MRI approach to quantify the ICA‐to‐ECA flow ratio (ratio_Q) as a surrogate of cerebrovascular resistance modulation within the respiratory band. We verified within‐session repeatability (Dataset_1) and examined physiology under free breathing and sustained deep breathing (Dataset_2). Respiration alone produced quantifiable changes in ratio_Q, with distinct amplitude and phase characteristics across conditions.

### 4.1 Quantifying Respiration‐Driven CRD With the ICA/ECA Flow Ratio (ratio_Q)

As outlined in Theory (Figure 1b), respiratory‐band carotid signals reflect both cardiac inflow (from intrathoracic‐pressure effects on cardiac output) and cerebrovascular resistance modulation; analyses based on cerebral blood flow oscillations alone cannot unambiguously attribute the source of these fluctuations. Accordingly, we use the ICA‐to‐ECA flow ratio (ratio_Q = Q_ICA/Q_ECA) to suppress global inflow effects via the synchronous ECA response and to highlight resistance‐related changes in the ICA.

Regarding flow quantification accuracy, RT‐PC requires a trade‐off between spatial and temporal resolution. While cerebral arterial flow can be accurately quantified under the selected spatiotemporal resolution [14], the ECA, owing to its smaller cross‐sectional area, is more susceptible to partial volume effects [22, 23], which may introduce inter‐individual variability in aver_ratio_Q related to differences in vessel size. Future studies may further mitigate partial volume effects by increasing spatial resolution while preserving high temporal resolution, for example through shared velocity encoding, compressed sensing, other accelerated imaging strategies, or hardware support with higher gradient performance.

Accurate identification of the respiratory frequency band is essential for isolating respiration‐driven CRD. Eliminating external respiratory belt recordings could simplify acquisition; however, under free‐breathing conditions, respiratory oscillations are less regular and lower in amplitude than during paced or deep‐breathing paradigms, complicating band definition [26, 27]. Venous flow, particularly in the IJV, exhibits stronger respiratory modulation than arterial signals and can therefore serve as an MRI‐derived reference [25, 28]. As shown in Figure S2, frequency‐domain comparisons between respiratory belt signals and right IJV flow in Dataset 2 demonstrate that belt‐derived spectra provide a clearer and less noise‐contaminated delineation of the respiratory band. Nevertheless, in most participants, the respiratory band remains identifiable from the IJV spectrum, supporting the general feasibility of IJV‐based respiratory‐band definition. Importantly, IJV flow oscillations exhibit variable phase offsets relative to respiration [25], precluding reliable reconstruction of respiratory‐cycle waveforms of Q_CA and ratio_Q from IJV alone. Overall, while IJV‐based respiratory‐band identification under free breathing is feasible, belt‐based recordings remain preferable. Future studies focusing on guided breathing or low‐frequency CRD analysis may omit belt recordings to streamline acquisition.

Methodological validation in Dataset_1 showed Excellent repeatability for aver_ratio_Q (ICC(3,1) = 0.96) and good repeatability for PI_ratio_Q (ICC(3,1) = 0.79; 95% CI 0.62–0.90); averaging three runs further improved agreement (ICC(3,k)). In Dataset 2, sustained deep breathing led to a significant 30% decrease in aver_Q_ICA, whereas aver_Q_ECA showed no significant change (a 5% increase with marginal significance, *p* = 0.094); respiration‐band magnitudes and PI increased for flow and for ratio_Q (Table 2).

Taken together, these results indicate that respiration induces dynamic modulation of global cerebrovascular resistance, which can be noninvasively and directly captured using the ICA‐to‐ECA flow ratio. Enabled by the high temporal resolution of real‐time phase‐contrast MRI, this approach provides the first quantitative evidence of cerebrovascular resistance oscillations at the respiratory frequency.

### 4.2 Respiration‐Driven CRD Contributes to Intracranial Flow Stability

CVR is essential for maintaining intracranial flow and volume homeostasis [20, 29]. Rather than reflecting stimulus‐evoked reactivity per se, respiration‐driven CRD describes intrinsic, time‐resolved modulation of vascular resistance that occurs across the breathing cycle. In the respiratory band, our data demonstrate that respiration‐driven CRD dynamically modulates carotid inflow across the breathing cycle and adjusts baseline resistance between conditions, thereby stabilizing intracranial hemodynamics under varying respiratory loads.

During free breathing, Q_CA increased during expiration while ratio_Q declined synchronously (Figures 6 and S1), indicating a transient increase in cerebrovascular resistance. This synchronous resistance rise counterbalanced transient Q_ICA increases, resulting in lower ICA flow pulsatility than ECA (PI_Q_ICA = 8.5% ± 2.6%, PI_Q_ECA = 15.8% ± 4.7%). Such respiration‐driven CRD effectively attenuated intracranial flow fluctuations within the respiratory cycle, thereby supporting intracranial volume stability.

Under sustained deep breathing, larger intrathoracic‐pressure swings markedly increase ampli_Q_CA and advance the Q_CA phase relative to free breathing (Figures 6 and S1). The inverse coupling between Q_CA and ratio_Q is reduced, suggesting reduced instantaneous vascular buffering of inflow changes. In parallel, aver_ratio_Q decreases (2.5 → 1.7; *p* < 0.001; dimensionless), consistent with an elevated mean cerebrovascular resistance. This reduction in aver_Q_ICA may represent a compensatory CRD‐mediated adjustment, helping to limit deep‐breathing–induced intracranial volume oscillations under increased respiratory load.

### 4.3 External Carotid Artery Compensation for ICA Flow Changes

Beyond intracranial regulation, respiration‐driven CRD also affects extracranial hemodynamics via the ECA, which supplies the face and other extracranial tissues. Prior work has reported distinctive oscillations in facial/skin blood flow—particularly in the low‐frequency (0.07–0.15 Hz; Mayer‐wave) range [21, 30]—though the mechanisms remain unclear.

With simultaneous ICA–ECA measurements, we observed that during free breathing the CRD‐related suppression of ICA oscillations was accompanied by fluctuations in ECA flow. Under sustained deep breathing, aver_Q_ICA decreased significantly whereas aver_Q_ECA remained stable (Table 2). Together, these findings are consistent with an ECA‐mediated redistribution when ICA flow is modulated by cerebrovascular resistance changes.

This interpretation provides a mechanistic context for the ECA oscillatory patterns reported previously and positions the ECA as a functional link between intracranial and extracranial circulations. Potential applications include extracranial vascular monitoring (e.g., postoperative facial transplantation surveillance [31]) and assessment of extracranial vascular disorders.

### 4.4 Respiration‐Driven Modulation of Cerebrovascular Resistance Dynamics

The present findings point to two complementary pathways by which respiration modulates CRD within the respiratory band.

Chemical pathway (transmission wave). Respiratory cycles induce fluctuations in arterial CO_2_—a potent vasoactive stimulus [1, 3, 5, 32]. After a transit delay of ˜6–12 s [33], these fluctuations reach the cerebral circulation and can elicit vasomotor responses [34]. Although CO_2_ was not directly measured in this study, sustained deep breathing is expected to increase CO_2_ variability; the observed rise in PI_ratio_Q under this condition is consistent with a chemical contribution.

Mechanical pathway (pressure wave). Each respiratory cycle alters intrathoracic pressure, instantaneously modulating cardiac output and thereby inducing oscillations in CBV, which are counterbalanced by opposite CSF shifts to maintain intracranial homeostasis [15, 16, 28]. Liu et al. [35] reported CBV oscillations of 0.47 ± 0.20 mL during free breathing and 1.44 ± 0.90 mL during sustained deep breathing, with CSF compensation of ˜70% and ˜95%, respectively. This pattern is consistent with upregulated vascular resistance during sustained deep breathing, limiting further CBV expansion and requiring more complete CSF compensation. In line with this mechanism, aver_ratio_Q decreased under sustained deep breathing.

Together, these observations support a dual‐pathway framework—delayed chemical feedback plus instantaneous mechanical loading—to interpret the phase and amplitude characteristics of Q_CA and ratio_Q across respiratory states. Within this framework, respiration‐driven CRD emerge as a key mediator linking respiratory mechanics to intracranial hemodynamic stability, while also providing a mechanistic bridge to CVR assessed using conventional paradigms.

### 4.5 Potential Applications and Method Extension

Quantification of CRD represents an important biomarker for neurological diseases [4, 7, 10, 36, 37]. As a resistance‐based and gas‐free framework, the present approach is simple and clinically feasible, requiring only spontaneous or instructed breathing without additional apparatus. Integration with motion correction further enhances robustness, reducing artifacts from patient movement.

Another key advantage lies in the use of the arterial flow ratio (ratio_Q), which directly reflects modulation of cerebrovascular resistance while minimizing confounding from cardiac‐driven fluctuations. This improves specificity over conventional flow‐based indices. Physiological activities such as sleep or anesthesia states [8, 38], postural changes (altering venous return and cerebral perfusion pressure) [39, 40], postprandial redistribution of blood flow (with mesenteric flow increasing 50%–100% at the expense of cerebral share) [41], and emotional stress (modulating cardiac output) [42] can markedly influence cerebral inflow. These fluctuations may confound flow‐based indices but do not necessarily reflect changes in cerebrovascular resistance. By filtering out such systemic effects, ratio_Q provides a more specific and reliable index of resistance modulation.

Beyond respiratory frequency, the method can also extend to lower spectral components. As illustrated in Figure S3, ratio_Q sensitively captured oscillations below 0.1 Hz during a single deep‐breathing maneuver, with resistance‐related fluctuations more prominent than those observed in ICA inflow. Importantly, even during ICA “plateaus” (segments c–d), ratio_Q continued to reveal ongoing resistance modulation, underscoring its ability to uncover subtle CRD that may remain obscured in conventional flow metrics.

Overall, ratio_Q offers a practical, dimensionless marker of cerebrovascular resistance modulation, with potential applications in bedside or longitudinal monitoring of CRD, particularly when gas challenges are impractical. By providing time‐resolved, resistance‐based information without gas challenges, this framework may also facilitate the development of faster, lower‐cost CVR assessment strategies and may support early detection of cerebrovascular disease.

### 4.6 Limitations and Prospectives

This study has several limitations.

First, ratio_Q at C2–C3 cannot resolve hemispheric or regional CRD because bilateral ICAs mix within the Circle of Willis; therefore, it reflects global resistance dynamics rather than spatially resolved CVR maps as obtained with CO_2_‐based BOLD fMRI [1, 12]. Nevertheless, this global, gas‐free RT‐PC approach should be viewed as a complementary alternative to conventional CVR methods. Future studies could use multi‐slice RT‐PC [43] to simultaneously acquire intracranial arterial flow (e.g., middle cerebral artery) together with ECA flow and compute region‐specific ratio_Q metrics, enabling regional or hemispheric characterization of CRD and their spatial heterogeneity.

Second, the modest cohort limited statistical power—particularly for sex‐, side‐, and age‐related (Figure S4) stratifications—so we reported distribution plots and simple nonparametric tests without deeper subgroup modeling. Prior studies based on low‐frequency cerebral perfusion signals have shown that females exhibit significantly higher CVR than males [44]; whether similar sex‐related differences extend to respiration‐driven CRD remains to be determined. Larger cohorts will therefore be required to enable a more comprehensive assessment of inter‐individual, sex‐related, and physiological variability in CRD.

Thirdly, the primary aim of this study was to establish a framework for quantifying respiration‐driven CRD and to address the methodological and baseline data gap within the respiratory band. Accordingly, we did not perform head‐to‐head comparisons with conventional gas‐challenge paradigms or other gas‐free indices. Future work may also explore direct comparisons between ratio_Q–based CRD metrics and cardiac‐cycle–based resistance indices, such as the Resistive Index [45], to disentangle respiratory‐ and cardiac‐scale vascular regulation and extend validation to lower‐frequency bands by comparison with established CVR modalities—including CO_2_ challenge, near‐infrared spectroscopy, and transcranial Doppler ultrasound—to evaluate sensitivity, specificity, and physiological correspondence across modalities [1, 5, 6, 9].

Finally, even under a CRD‐based interpretation, ratio_Q should be regarded as a resistance‐related proxy rather than a direct or exclusive measure of cerebrovascular resistance. While it captures respiration‐driven modulation of cerebrovascular resistance, ratio_Q may still be influenced by vascular compliance as well as other systemic or local physiological factors. Further work is therefore required to disentangle these contributions, including validation across complementary modalities and extended analyses across frequency bands.

## 5 Conclusion

The ICA‐to‐ECA flow ratio (ratio_Q) reduces cardiac‐related inflow interference, providing a stable and physiologically specific measure of respiration‐driven CRD. The proposed method demonstrated high reproducibility across repeated tests, supporting its reliability in both research and clinical settings.

With this approach, we validated and quantified respiration‐driven CRD, confirming its role in stabilizing intracranial flow fluctuations under different breathing conditions. In addition, we identified that the external carotid artery acts as a complementary pathway for flow redistribution accompanying internal carotid artery inflow changes under respiration‐driven CRD.

These findings establish ratio_Q as a simple, robust, and clinically viable biomarker of CRD, offering new insights into respiratory‐linked vascular regulation. By enabling time‐resolved, resistance‐based assessment without gas challenges, this approach provides a methodological foundation for the development of faster and lower‐cost CVR evaluation strategies, with potential applications in early diagnosis, longitudinal monitoring, and treatment assessment of cerebrovascular disorders.

## Funding

This research was funded by the France National Research Agency (Agence Nationale de la Recherche) (reference: CALCOCIMO ANR‐23‐CE18‐0026 and EquipEX FIGURES 10‐EQPX‐0001).

## Supporting information


**Figure S1:** Reconstructed respiratory‐cycle waveforms of Q_CA and ratio_Q during free and deep breathing. (a) Free‐breathing condition. Normalized reconstructed respiratory‐cycle waveforms of total carotid inflow (Q_CA) and flow ratio (ratio_Q) are shown separately for the right and left sides. Thin gray lines represent individual subjects, while colored curves indicate group‐averaged waveforms. (b) Deep‐breathing condition. Corresponding reconstructed respiratory‐cycle waveforms for Q_CA and ratio_Q during sustained deep breathing, displayed using the same conventions as in (a). For both breathing conditions, all waveforms were normalized to each subject's mean value (dimensionless). The *x*‐axis represents the respiratory cycle (0%–100%), with expiration and inspiration phases indicated.
**Figure S2:** Frequency‐domain representation of respiratory belt and right internal jugular vein (IJV‐R) flow signals during free breathing in Dataset 2 (*N* = 17). (a) Representative example from subject T17. Time‐domain signals of the respiratory belt (top, gray) and right internal jugular vein flow (IJV‐R; bottom, blue) were transformed into the frequency domain using fast Fourier transform (FFT). Red dashed lines denote the respiratory‐frequency band defined based on the respiratory belt signal, while the blue curve shows the spectral amplitude of the IJV‐R flow signal. (b) Frequency‐domain representations of the respiratory belt signal (gray) and IJV‐R flow signal (blue) for the remaining 16 participants, displayed over the 0.1–0.9 Hz frequency range.
**Figure S3:** Example illustrating CVR dynamics at + low frequencies (< 0.1 Hz) during a 1‐min RT‐PC acquisition. The participant performed a single deep breath from the 6th second, followed by a return to normal breathing. Low‐frequency components (< 0.1 Hz) of ICA (Q_ICA, red) and ECA (Q_ECA, purple) flow signals were extracted by low‐pass filtering. Total carotid artery flow (Q_CA, black) and flow ratio (ratio_Q, cyan) were calculated. The phases indicating increased and decreased cerebrovascular resistance are marked, demonstrating the sensitivity of the method for tracking subtle, transient CVR changes induced by respiratory maneuvers.
**Figure S4:** Spearman correlation matrices between age and flow‐derived hemodynamic and CRD‐related parameters. Spearman rank correlation matrices are shown for right‐sided (left panel) and left‐sided (right panel) parameters, computed across the combined cohort (*n* = 27; Dataset 1 + Dataset 2). Color and ellipse orientation indicate the direction and magnitude of the correlation coefficient (ρ), with red denoting positive and blue denoting negative correlations. Statistical significance is indicated as *p* < 0.05 (*), *p* < 0.01 (**), and *p* < 0.001 (***). These analyses are exploratory and intended to illustrate age‐related associations across flow‐, pulsatility‐, and ratio‐based metrics.

## Data Availability

The raw DICOM data cannot be shared publicly due to institutional and national privacy regulations. De‐identified processed flow data are available from the corresponding author upon reasonable request. The post‐processing software used in this study is under preparation for public release (including full user documentation). In the meantime, access to the software can be provided upon request by contacting Pan Liu (P.L.) or Olivier Balédent (O.B.).
