# Supplementary material for: Respiration Can Trigger Cerebrovascular Reactivity: A Novel Method to Quantify Cerebrovascular Resistance Dynamics Using Real‐Time Phase‐Contrast MRI
Source: Magn Reson Med. 2026 Feb 25;96(1):83–95. doi: 10.1002/mrm.70322 (PMC13156442; doi:10.1002/mrm.70322)
Supplement: Supplementary file 1 — Figure S1: Reconstructed respiratory‐cycle waveforms of Q_CA and ratio_Q during free and deep breathing. (a) Free‐breathing condition. Normalized reconstructed respiratory‐cycle waveforms of total carotid inflow (Q_CA) and flow ratio (ratio_Q) are shown separately for the right and left sides. Thin gray lines represent individual subjects, while colored curves indicate group‐averaged waveforms. (b) Deep‐breathing condition. Corresponding reconstructed respiratory‐cycle waveforms for Q_CA and ratio_Q during sustained deep breathing, displayed using the same conventions as in (a). For both breathing conditions, all waveforms were normalized to each subject's mean value (dimensionless). The x‐axis represents the respiratory cycle (0%–100%), with expiration and inspiration phases indicated. Figure S2: Frequency‐domain representation of respiratory belt and right internal jugular vein (IJV‐R) flow signals during free breathing in Dataset 2 (N = 17). (a) Representative example from subject T17. Time‐domain signals of the respiratory belt (top, gray) and right internal jugular vein flow (IJV‐R; bottom, blue) were transformed into the frequency domain using fast Fourier transform (FFT). Red dashed lines denote the respiratory‐frequency band defined based on the respiratory belt signal, while the blue curve shows the spectral amplitude of the IJV‐R flow signal. (b) Frequency‐domain representations of the respiratory belt signal (gray) and IJV‐R flow signal (blue) for the remaining 16 participants, displayed over the 0.1–0.9 Hz frequency range. Figure S3: Example illustrating CVR dynamics at + low frequencies (< 0.1 Hz) during a 1‐min RT‐PC acquisition. The participant performed a single deep breath from the 6th second, followed by a return to normal breathing. Low‐frequency components (< 0.1 Hz) of ICA (Q_ICA, red) and ECA (Q_ECA, purple) flow signals were extracted by low‐pass filtering. Total carotid artery flow (Q_CA, black) and flow ratio (ratio_Q, cyan) were calculated. [file MRM-96-83-s001.docx]

Supplementary

## S1. Reconstructed respiratory-cycle waveforms of Q_CA and ratio_Q under free and deep breathing.


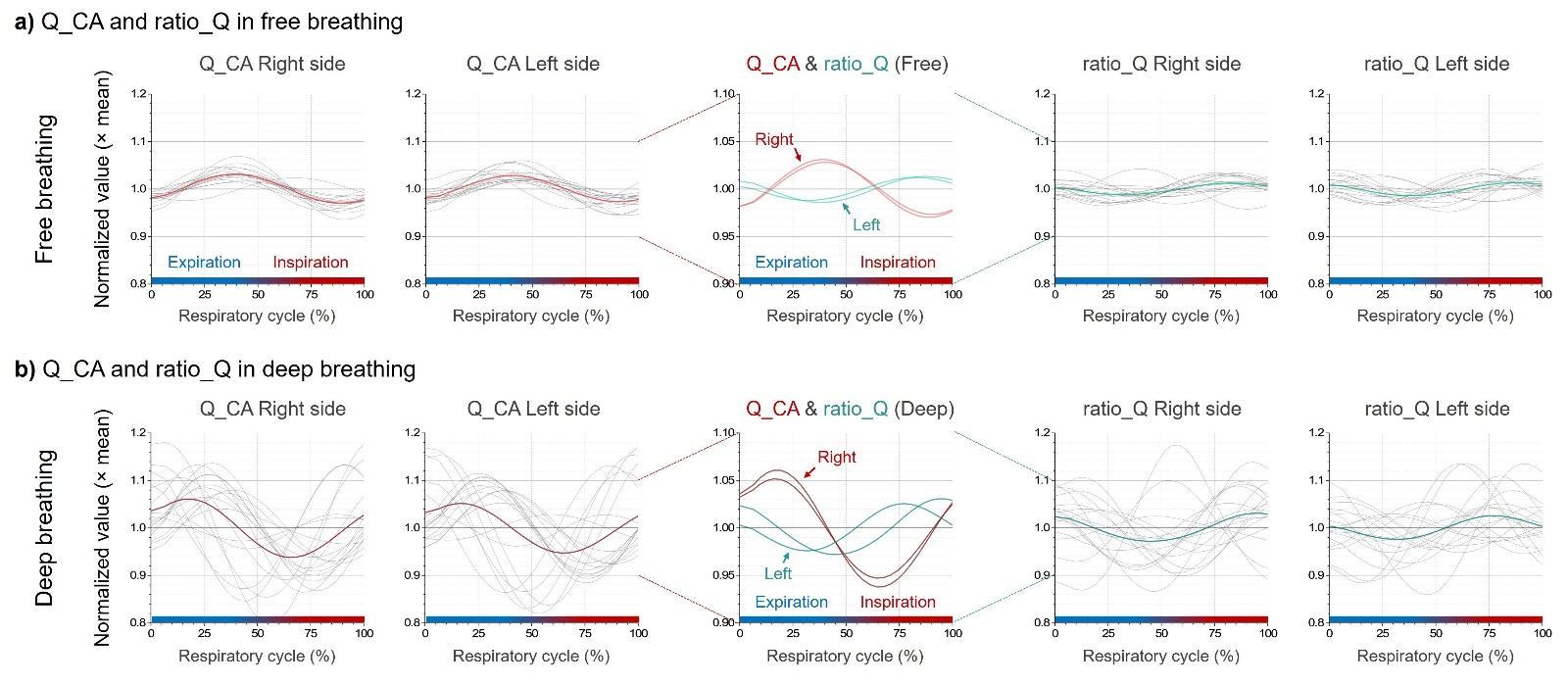


Figure S1 **Reconstructed respiratory-cycle waveforms of Q_CA and ratio_Q during free and deep breathing. a)** Free-breathing condition. Normalized reconstructed respiratory-cycle waveforms of total carotid inflow (Q_CA) and flow ratio (ratio_Q) are shown separately for the right and left sides. Thin gray lines represent individual subjects, while colored curves indicate group-averaged waveforms. **b)** Deep-breathing condition. Corresponding reconstructed respiratory-cycle waveforms for Q_CA and ratio_Q during sustained deep breathing, displayed using the same conventions as in (a). For both breathing conditions, all waveforms were normalized to each subject’s mean value (dimensionless). The x-axis represents the respiratory cycle (0–100%), with expiration and inspiration phases indicated.

## S2. Feasibility analysis of respiratory-band identification using internal jugular vein flow.

**
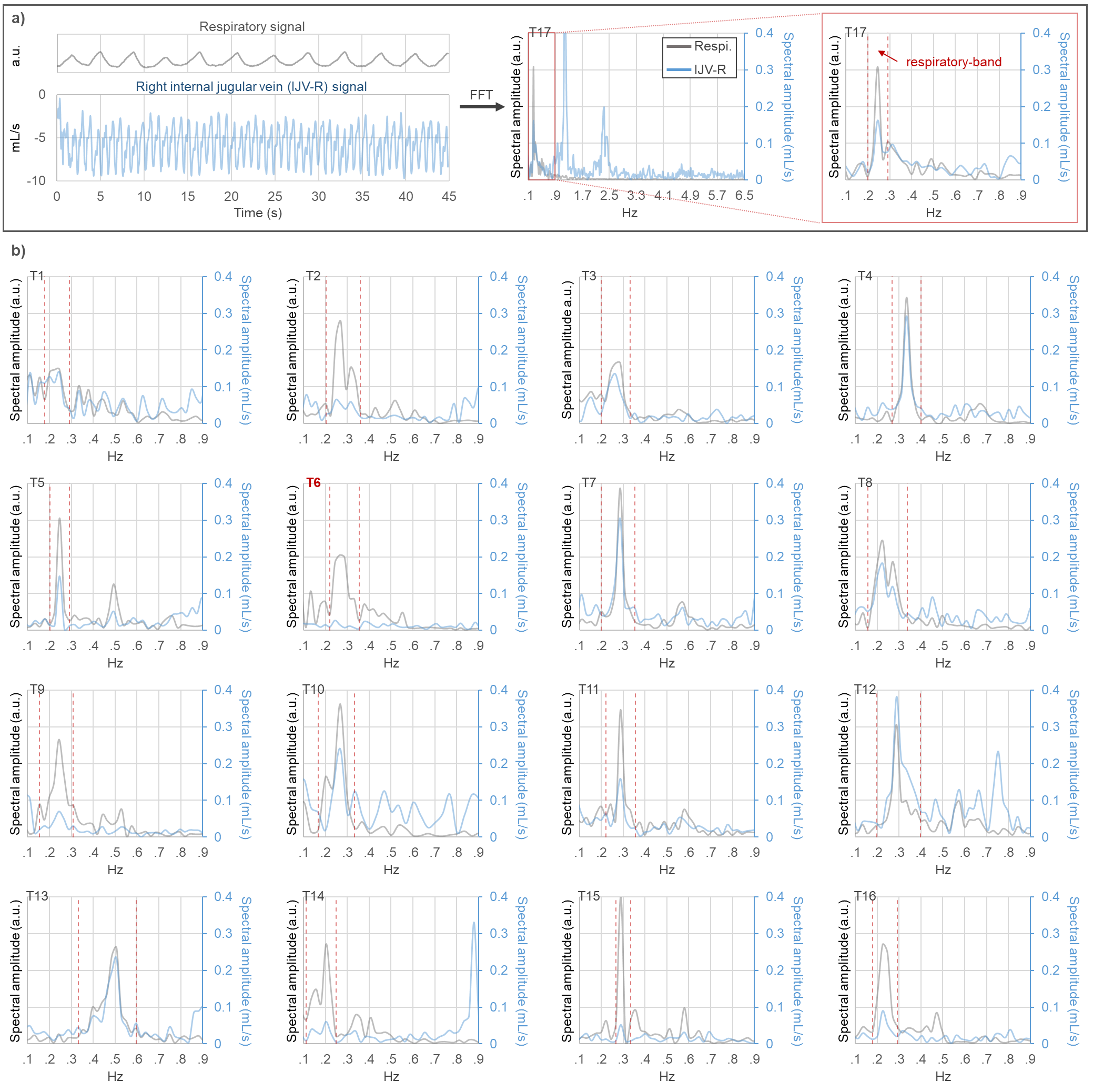
**

Figure S2 **Frequency-domain representation of respiratory belt and right internal jugular vein (IJV-R) flow signals during free breathing in Dataset 2 (N = 17). a)** Representative example from subject T17. Time-domain signals of the respiratory belt (top, gray) and right internal jugular vein flow (IJV-R; bottom, blue) were transformed into the frequency domain using fast Fourier transform (FFT). Red dashed lines denote the respiratory frequency band defined based on the respiratory belt signal, while the blue curve shows the spectral amplitude of the IJV-R flow signal. **b)** Frequency-domain representations of the respiratory belt signal (gray) and IJV-R flow signal (blue) for the remaining 16 participants, displayed over the 0.1–0.9 Hz frequency range.

Under free-breathing conditions, respiratory-band oscillations in the internal jugular vein (IJV) are generally weaker and less regular than those observed during paced or deep-breathing paradigms, making respiratory-band identification intrinsically more challenging.

Nevertheless, comparison between the IJV flow spectra and the respiratory belt spectra indicates that, for the majority of participants, the respiratory frequency band can still be reasonably identified from the IJV signal. Compared with belt-derived spectra, IJV spectra are more susceptible to interference from other frequency components, including low-frequency fluctuations and cardiac-related harmonics, and typically exhibit a higher background noise level. As a result, the respiratory-band component is less prominent, and in some participants (e.g., T2 and T6), the respiratory peak is not clearly distinguishable.

Overall, defining the respiratory frequency band solely from IJV flow under free-breathing conditions is less robust than using a respiratory belt signal, but remains generally feasible and may serve as an alternative reference when external respiratory recordings are unavailable.

## S3. Application of the proposed method to quantify cerebrovascular reactivity (CVR) at very-low frequencies (<0.1 Hz) triggered by a single deep breath.


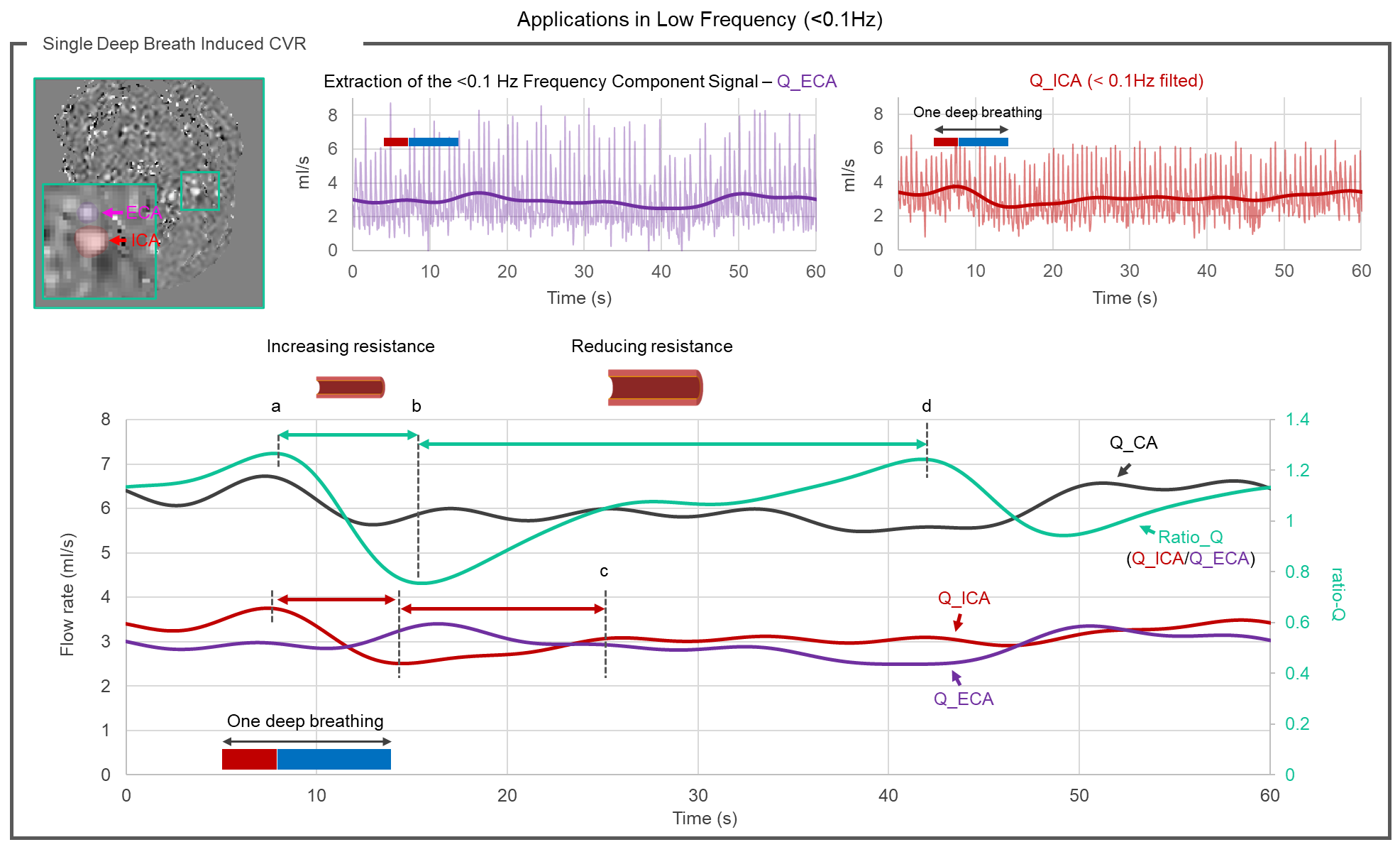


Figure S3 **Example illustrating CVR dynamics at+ low frequencies (<0.1 Hz) during a one-minute RT-PC acquisition.** The participant performed a single deep breath from the 6th second, followed by a return to normal breathing. Low-frequency components (<0.1 Hz) of ICA (Q_ICA, red) and ECA (Q_ECA, purple) flow signals were extracted by low-pass filtering. Total carotid artery flow (Q_CA, black) and flow ratio (ratio_Q, cyan) were calculated. The phases indicating increased and decreased cerebrovascular resistance are marked, demonstrating the sensitivity of the method for tracking subtle, transient CVR changes induced by respiratory maneuvers.

Figure S3 demonstrates that the proposed RT-PC approach effectively captures cerebrovascular reactivity (CVR) dynamics at very-low frequencies (<0.1 Hz), extending its utility beyond respiratory frequency analysis (PI_ratio_Q). In this example, ratio_Q sharply decreases several seconds after initiating a single deep breath (segment a–b), indicating increased cerebrovascular resistance. Notably, during this phase, the opposing changes between Q_ICA and Q_ECA clearly illustrate compensatory external carotid flow enhancement as internal carotid flow diminishes, underscoring the sensitivity and specificity of ratio_Q compared to Q_ICA alone. Furthermore, the interval c–d highlights that cerebrovascular resistance can fluctuate substantially even when Q_ICA appears relatively stable, supporting ratio_Q as a more sensitive metric for detecting subtle CVR changes.

## S4. Spearman correlation matrices between age, hemodynamic variables, and CRD-related parameters (n = 27).


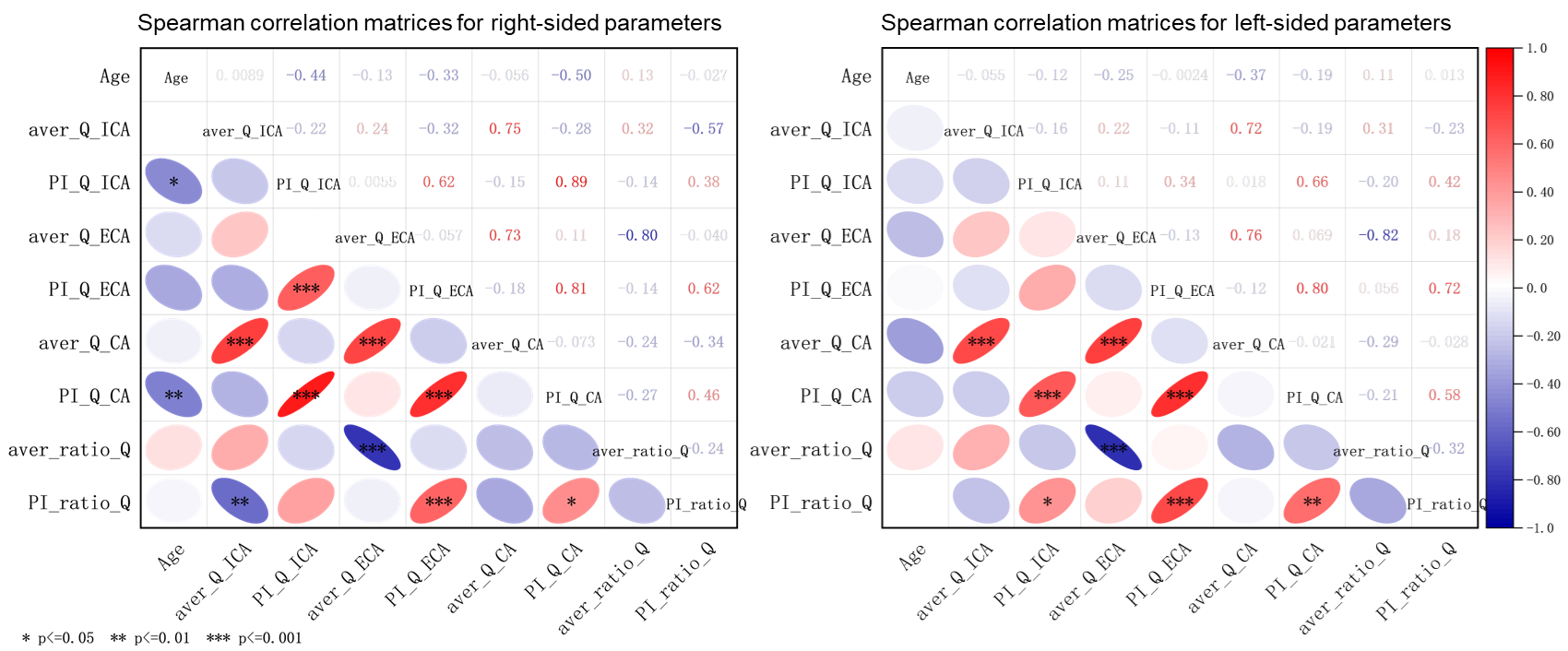


Figure S4 **Spearman correlation matrices between age and flow-derived hemodynamic and CRD-related parameters.** Spearman rank correlation matrices are shown for right-sided (left panel) and left-sided (right panel) parameters, computed across the combined cohort (n = 27; Dataset 1 + Dataset 2). Color and ellipse orientation indicate the direction and magnitude of the correlation coefficient (ρ), with red denoting positive and blue denoting negative correlations. Statistical significance is indicated as *p* < 0.05 (**), p < 0.01 (******),*** *and p < 0.001 (****). These analyses are exploratory and intended to illustrate age-related associations across flow-, pulsatility-, and ratio-based metrics.

Given the limited sample size and the exploratory nature of the analysis, these observations are presented for descriptive purposes only and are not intended for inferential conclusions.

The overall correlation patterns were broadly similar between the right and left sides.

Age showed a tendency to be associated with flow-based pulsatility indices; however, no clear age dependence was observed for ratio_Q–based metrics.

It should also be noted that this correlation analysis combines data acquired on two different MRI systems, which may introduce additional variability related to hardware performance and sequence implementation. Future studies with larger, age-balanced cohorts acquired on a single platform will be required to more robustly assess age-related effects.

Beyond cross-sectional associations, future work may benefit from incorporating additional respiratory paradigms and transition phases (e.g., sustained deep breathing followed by return to free breathing) to develop CRD-based quantitative protocols, such as characterizing the recovery dynamics of CRD following respiratory challenges, and to further investigate age-related modulation of cerebrovascular regulation.
